# The enigmatic SLC26A6 multifunctional anion transporter: recent advances in structure-function relationship, pathophysiological significance and novel pharmacological inhibitors

**DOI:** 10.3389/fphar.2024.1536864

**Published:** 2025-01-30

**Authors:** Ursula E. Seidler

**Affiliations:** Department of Gastroenterology, Hannover Medical School, Hanover, Germany

**Keywords:** anion exchange, cryo-microscopy, SLC26 family, hyperuricemia, nephrolithiasis, pancreatic secretion, acid base balance, intestine

## Abstract

SLC26A6, a member of the SLC26 family of multifunctional anion transporters, has been particularly enigmatic because of its multiple modes of transport, its expression in organs that are difficult to study physiologically, and the lack of specific antibodies and inhibitors. This has recently changed. SLC26A6 is expressed in the human pancreas, kidney, intestine, heart and some other organs and is involved in fluid absorption, anion secretion, regulation of intracellular pH and elimination of waste products such as oxalate. This review will focus on three topics: Firstly, a molecular structure of human SLC26A6 has recently been obtained by cryo-electron microscopy. Structure-function studies of the reconstituted SLC26A6 in proteoliposomes suggested a 1:1 stoichiometry, resulting in electroneutral Cl^−^/HCO_3_^−^ exchange and electrogenic Cl^−^/oxalate^2−^ exchange. How do these data help to understand the published functional studies? Secondly, whole exon sequencing of a kidney stone cohort from the United Kingdom database revealed a dominant negative SLC26A6 mutation in a patient with enteric hyperoxaluria, oxalate kidney stones and a low calcium diet. How does this finding fit with previous genetic studies in mice and humans of SLC26A6 gene mutations? Thirdly, progress has been made in identifying specific inhibitors for SLC26A6. Where might this be of clinical relevance?

## Brief summary of SLC26A6 cloning, expression and transport studies

Solute carrier family 26 (SLC26) is a family of functionally diverse anion transporters found in all kingdoms of life. The human genome encodes 10 functional homologs, several of which are causally associated with major human diseases. The SLC26A6 gene from different species has been cloned in parallel by several researchers and has also been named CFEX and PAT-1 (Lohi et al., 2000; Knauf et al., 2001; Waldegger et al., 2001). SLC26A6 appears to be able to transport a wide range of mono- and divalent anions, including formate, sulphate, oxalate, nitrate, Cl^−^ and HCO_3_^−^. SLC26A6 mRNA and protein expression was first reported in the pancreas (Lohi et al., 2000), the kidney (Knauf et al., 2001; Waldegger et al., 2001) and the intestine (Wang et al., 2002). The mRNA is found in many other organs, while the immunohistochemical data are less clear due to nonspecific binding of many anti-SLC26a6 antibodies (Seidler and Nikolovska, 2019; Wang et al., 2020). Slc26a6-deleted mice showed alterations in epithelial transport functions in the pancreatic ducts (Song et al., 2012), in the proximal intestine (Wang et al., 2005; Tuo et al., 2006; Singh et al., 2008a; Singh et al., 2008b; Singh et al., 2010; Knauf et al., 2011; Xia et al., 2014; Neumeier et al., 2020), and the salivary glands (Shcheynikov et al., 2008), without morphological abnormalities, as well as the development of kidney stone on a high oxalate diet (Jiang et al., 2006), and deficits in pH_i_-regulation in the heart (Sirish et al., 2017).

Early transport studies in *Xenopus* oocytes and in HEK293 cells by different groups suggested different stoichiometries, and therefore electroneutral vs. electrogenic Cl^−^/HCO_3_^−^ exchange (Ko et al., 2002; Chernova et al., 2005; Shcheynikov et al., 2006). Because the aminoacid sequence similarity is unusally low between the human and mouse orthologs of SLC26A6 with 78% identity, functional studies have compared their transport characteristics when expressed in *Xenopus* oocytes: While human SLC26A6-mediated electroneutral (2?)Cl^−^/oxalate exchange^2−^, the exchange by murine Slc26a6 appeared electrogenic (Jiang et al., 2002; Chernova et al., 2005; Clark et al., 2008). The native tissue transport studies could not resolve these controversies, because Slc26a6 is coexpressed with, and its transport activity influenced by, many other transporters, which may influence the electrogenicity or–neutrality of a studied transport process. Since comprehensive reviews discuss these early experiments in detail (Ohana et al., 2009; Aronson, 2010; Ishiguro et al., 2012; Alper and Sharma, 2013; Ohana, 2015; Yamaguchi et al., 2017; Seidler and Nikolovska, 2019; Wang et al., 2020), I will focus on the recently published structure-function studies in reconstituted liposomes, on human disease causing mutations, and recent pharmacological developments.

## Identification of the molecular structure of SLC26 family members

**FIGURE 1:**
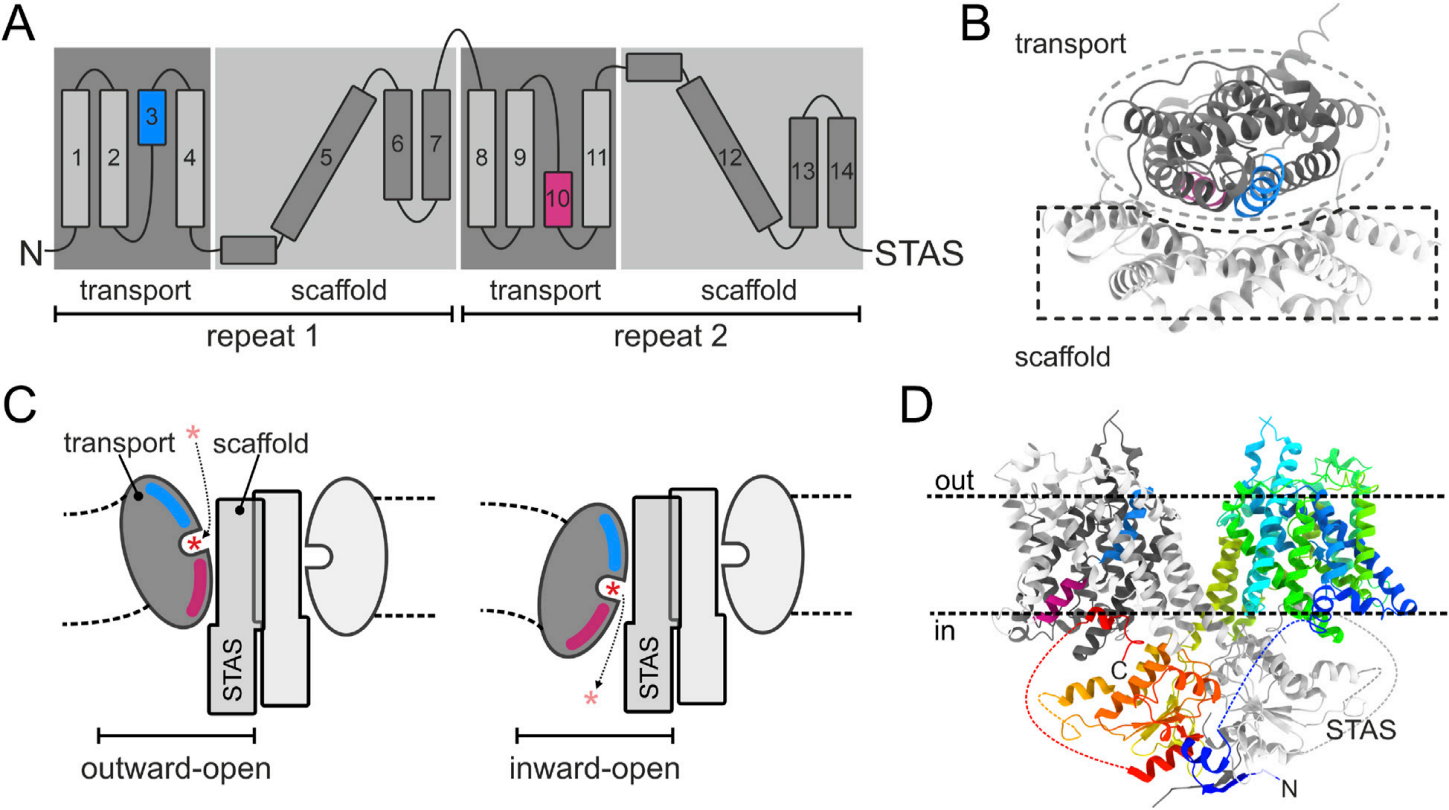
Structure and transport mechanism common for the SLC26A family. **(A)** Topology diagram of SLC26 proteins. TM3 and TM10, which form the substrate binding site, are colored blue and pink, respectively. **(B)** Membrane domain of a schematic SLC26A protomer viewed from the outside on the membrane. Transport and scaffold domains are shown in dark and light gray, respectively. **(C)** Side view in the plane of the membrane at the interface of the transport and scaffold domains, showing the opening of the SLC26A isoform to the extracellular space and the cytoplasm, respectively. The expected deformation of the lipid membrane as a result of the elevator movement of the transport domain is indicated. Asterisk indicates the transported anion. **(D)** Side view in the plane of the membrane on the human SLC26A9 dimer. One protomer is shown in a rainbow color scheme with the N- and C-terminus in blue and red, respectively. The opposite protomer is shown in the color scheme of panel **(B)**. Dotted lines indicate intervening sequences (not structurally resolved) in the STAS domain.

The first member of the SLC26 family whose crystal structure was identified, was SLC26Dg, a prokaryotic proton-coupled fumarate transporter (Geertsma et al., 2015). SLC26Dg shares 46% residue similarity in the transmembrane (TM) region with human Prestin and 57% with the *Escherichia coli* transporter DauA. When reconstituted in lipid bilayers, the purified SLC26Dg forms a homodimer, which had already been predicted from previous studies for the whole family (Detro-Dassen et al., 2008; Compton et al., 2014). Using a specific method for protein expression and an anti-SLC26Dg nanobody to facilitate crystallisation (Chang and Geertsma, 2017), the group was able to crystallize SLC26Dg monomers to a resolution of 3.2 Å (Geertsma et al., 2015). The membrane-inserted domain consisted of two intertwined inverted repeats of seven transmembrane segments each (Figure 1A). The transmembrane part of SLC26Dg is organised into two parallel subdomains, with the helices 1–4 and 8–11 folding into a compact unit designated as the “transport” or “core” domain, and the Helices 5–7 and 12–14 forming an elongated structure called the “scaffold” or “gate” domain, which shields one side of the transport domain (Figure 1B). The anion binding site was proposed to be located in the center of the transport domain, close to the scaffold domain, and transport to occur by an “elevator-like” mechanism, in which the mobile transport domain moves against the rigid scaffold domain (Figure 1C). One of the best supporting experimental evidence for this “elevater mechanism” was provided by the groups of Jan-Philipp Machtens and Dominik Oliver during their studies on the transport mode of prestin (SLC26A5) (Kuwabara et al., 2023).

This mode of transport was assumed to be representative for the whole SLC26 family, and this has so far been proven correct, with structure information having been published for several more of the mammalian, as well as prokaryotic members of the family (Wang et al., 2019; Chi et al., 2020; Bavi et al., 2021; Wang et al., 2021; Ge et al., 2021; Butan et al., 2022; Futamata et al., 2022; Li et al., 2023; Liu et al., 2023; Tippett et al., 2023; Wang et al., 2024). The first mammalian SLC26 member, for which a high resolution structure was published, was the murine Slc26a9 (Walter et al., 2019), shortly later followed by publication of the human homolog (Chi et al., 2020). This revealed the structure of the complete dimeric SLC26 assembly, showing that each membrane domain interacts with the cytoplasmatic Sulfate Transporter and Anti-Sigma factor antagonist (STAS) domain of the opposite protomer (Figure 1D).

## Structure-function studies of human SLC26A6

**FIGURE 2:**
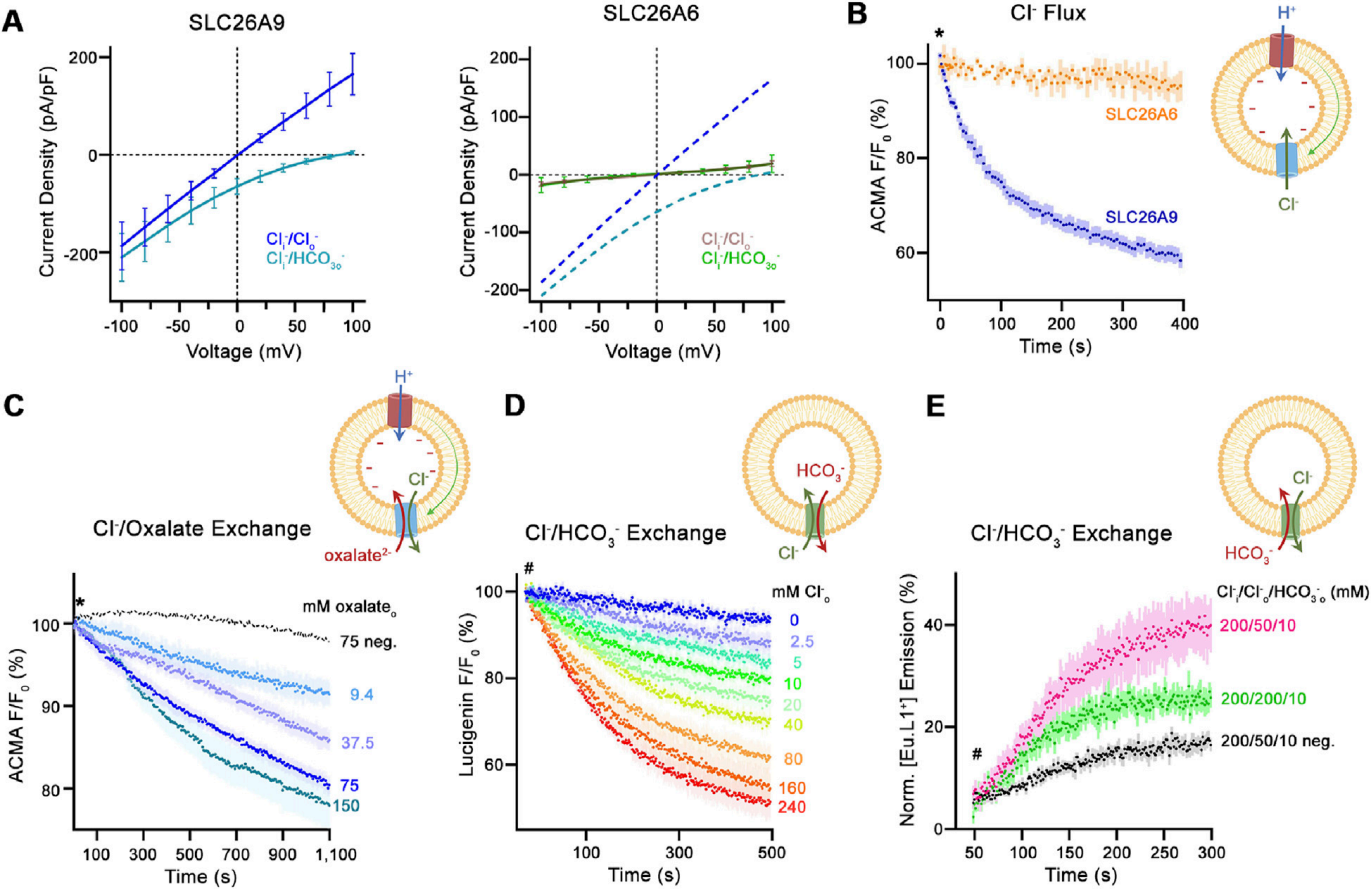
Transport properties of SLC26A6. **(A)** Current-Voltage relationships of HEK 293 cells expressing murine SLC26A9 (left graph) and human SLC26A6 (right graph). **(B)** Uncoupled Cl^−^ transport mediated by either the modified murine construct SLC26A9T or SLC26A6 reconstituted into proteoliposomes, as monitored by the fluorescence change of the pH-sensitive fluorophore ACMA. Asterisk indicates addition of the protonophore CCCP, which allows counterion movement and electrogenic Cl^−^ transport to proceed. **(C)** Electrogenic oxalate uptake followed by the fluorescence change of the pH-sensitive fluorophore ACMA. Traces show mean quenching of ACMA fluorescence in a time- and concentration-dependent manner for SLC26A6 proteoliposomes. Asterisk indicates addition of the protonophore CCCP, which allows counterion movement and electrogenic Cl^−^ transport to proceed. **(D)** Coupled Cl^−^/HCO_3_^−^ exchange monitored by the time- and concentration-dependent quenching of the fluorophore lucigenin trapped inside proteoliposomes containing SLC26A6. Hashtag indicates the addition of the assayed anion. **(E)** Coupled Cl^−^/HCO_3_^−^ exchange monitored by the time- and concentration-dependent luminescence increase of the HCO_3_^−^-selective probe [Eu.L1+] trapped inside proteoliposomes containing SLC26A6. Hashtag indicates the addition of the assayed anion. For details regarding the methods and experimental protocol see Tippett et al. (2023).

The same group that analysed the structure and transport properties of mouse Slc26a9 (Walter et al., 2019) has recently published the functional and structural analysis of the human SLC26A6 isoform (Tippett et al., 2023). These very informative experiments will now be reviewed in detail: The group first expressed human SLC26A6 and murine Slc26a9 in HEK293 cells and performed whole-cell patch-clamp experiments, assuming that the reported electrogenic SLC26A6 (Shcheynikov et al., 2006; Ohana et al., 2009) could be studied by establishing a current-voltage relationship similar to that observed for Slc26a9. However, in contrast to Slc26a9, no specific current was measured at any voltage (Figure 2A), despite the fact that the membrane expression of SLC26A6 and Slc26a9 was similar. The group then expressed, purified and reconstituted the protein in proteoliposomes. The transport function was assessed using different fluorophores, always in comparison with that of Slc26a9t (a Slc26a9 with truncated IVS sequences for better membrane insertion). Using the pH-sensitive dye ACMA and the protonophore CCCP, the researchers were able to identify electrogenic anion influx. Electrogenic anion influx leads to the build-up of a negative potential. In the presence of the protonophore CCCP, this results in proton influx, reducing the pH inside the liposomes and quenching the ACMA fluorescence. In contrast to Slc26a9t-reconstituted proteoliposomes, external Cl^−^ did not induce ACMA fluorescence quenching in SLC26A6-reconstituted proteoliposomes (Figure 2B). However, external oxalate (Ox^2−^) resulted in ACMA quenching in Cl^−^ loaded SLC26A6-reconstituted proteoliposomes, suggesting that SLC26A6-mediated Ox^2−^/Cl^−^ exchange is electrogenic (Figure 2C).

SLC26A6-mediated Cl^−^_o_/HCO_3_^−^_i_ exchange was assessed by measuring Cl^−^ uptake into HCO_3_^−^- loaded proteoliposomes using the Cl^−^-sensitive dye lucigenin (Figure 2D). SLC26A6-mediated Cl^−^_i_/HCO_3_^−^_o_ exchange was assessed by loading the proteoliposomes with the novel HCO_3_^−^-sensitive europium probe [Eu.Lv1^+^] (Figure 2E). In conclusion, the results suggested that SLC26A6 mediates electroneutral Cl^−^/HCO_3_^−^ exchange, and electrogenic Ox^2−^/Cl^−^ exchange.

## Molecular basis for the strikingly different transport modes of SLC26A9 and SLC26A6

**FIGURE 3:**
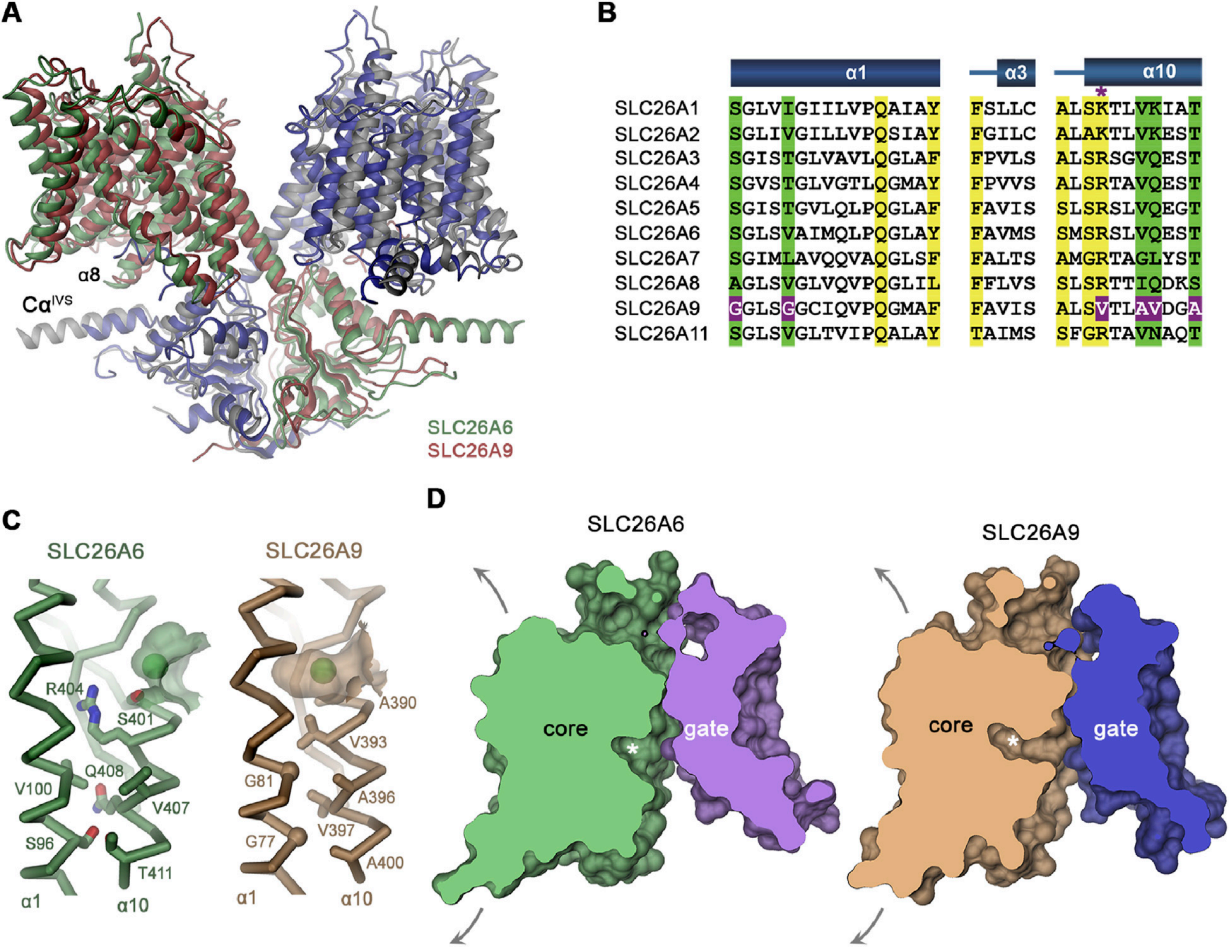
Comparison of the molecular structure of SLC26A9 and SLC26A6. **(A)** Ribbon representation of the superimposed SLC26A6 (green, gray) and SLC26A9 (red, blue, PDBID: 7CH1) dimers **(B)** Sequence alignment of the region constituting the ion binding site of the ten functional human SLC26 paralogs. Conserved residues in the contact region between α1 and α10 are highlighted in green, residues involved in ion interactions in yellow. Deviating residues in SLC26A9 are highlighted in violet. Asterisk marks position that harbors a basic residue in all family members except for SLC26A9, where the residue is replaced by a valine. Whereas most paralogs, including the ones operating as HCO_3_^−^ exchangers, carry an arginine at this site, the sulfate transporters SLC26A1 and 2 contain a smaller lysine. **(C)** Cα-representation of the contact region between α1 and α10 of **(C)** SLC26A6 and SLC26A9. The green ball represents the anion in the binding pocket **(D)**. Slice across a surface of the TM domains of SLC26A6 **(C)** and SLC26A9 **(D)** viewed from within the membrane. The spacious aqueous cavity leading to the ion binding site from the cytoplasm is evident. Asterisk indicated the position of the transported ion. Arrows indicate possible movements of the core domain. For details, see Tippett et al. (2023).

The molecular structure of SLC26A6 was determined by cryo-electron microscopy in an inward-facing conformation. A data set of the purified protein at 3.3 Å allowed unambiguous interpretation by an atomic model. Although the transport mode of SLC26A9 and LC26A6 differ remarkably, the overall molecular structures of SLC26A6 and SLC26A6 were found to be quite similar (Figure 3A), (Tippett et al., 2023). This is also true for the other mammalian members of the SLC26A family whose structure was recently identified, implying a common molecular mechanisms underlying their diverse transport functions (Wang et al., 2019; Chi et al., 2020; Bavi et al., 2021; Wang et al., 2021; Ge et al., 2021; Butan et al., 2022; Futamata et al., 2022; Li et al., 2023; Liu et al., 2023; Tippett et al., 2023; Wang et al., 2024). The scaffold (gate) domains of SLC26A9 (Chi et al., 2020) and SLC26A (Tippett et al., 2023) form a rigid structure in the dimer together with the cytoplasmic STAS domains, which remain relatively static during transport. Since the STAS domains contain intervening sequences whose molecular structures are yet unresolved, it is not clear how much movement occurs during the transport process. The scaffold domains do not show pronounced differences between the two paralogs. However, the anion binding sites in the centre of the transport (core) domain facing the gate domain showed distinctly different features between SLC26A9 and SLC26A6. These differences were largely confined to the conformation of the α-helix 10, which forms direct interactions with the transported ions. A comparison of the amino acid sequence lining this short sequence of α10, as well as that of the side chains of α1 and α3 facing the binding pocket, revealed several amino acid substitutions (Figure 3B). Modelling of these different amino acids suggested that the anion binding pocket is considerably deeper in SLC26A9 compared to SLC26A6 (Figures 3C, D). The large arginine in R404, which is also present in most other SLC26 paralogs (all that are known to accept HCO_3_^−^ in their binding site), but replaced by a valine in SLC26A9, interacts with the residues preceding α3, resulting in a more shallow pocket, and in addition likely also directly interacts with HCO_3_^−^ and Ox^2−^.

In order to test this hypothesis, the investigators mutated the R404 to the amino acid found in SLC26A9. SLC26A6 mutant R404V did not interfere with the protein integrity, and displayed good expression levels in HEK293 cells. Since whole-cell Cl^−^ currents were detectable but low compared to SLC26A9, the SLC26A6 mutant R404V was reconstituted into proteoliposomes. Using the pH-sensitive ACMA dye in the presence of the CCCP protonophore, the SLC26A6 mutant R404V displayed considerable uncoupled Cl^−^ conductance. The Cl^−^/HCO_3_^−^ exchange activity, assayed by loading the proteoliposomes with HCO_3_^−^ and with the Cl^−^ sensitive dye lucigenin, on the other hand, was markedly reduced. These data underpinned the importance of the Arg 404 for anion interactions and coupling, but also demonstrated that converting a SLC26 member with anion exchange characteristics into a fast Cl^−^ conductor requires more structural changes that mutating a single amino acid residue.

Also likely to be involved in transport regulation, but of unknown importance, are the structurally not resolved regions of the “intervening sequence” that links secondary structure elements of the STAS domain. They may be important platforms for interaction with other proteins or with the inner leaflet of the plasma membrane, thus influencing many aspects of anion transport regulation. We do not currently know much about the transport regulation of SLC26A6, but knowledge of the structural basis of these proteins will help us to explore this topic (Tippett et al., 2023).

At the identical time of the publication of the structure-function studies of SLC26A6, the murine Slc26a4 molecular structure has been resolved (Liu et al., 2023). As predicted for all mammalian SLC26 members, Slc26a4 formed homodimers with the anion binding pocket between TMD3 and TMD10. As for SLC26A6, symmetric homodimers in an inwardly facing open state were visualized when the SLC26a4 molecules were frozen in either Cl^−^ or in HCO_3_^−^ solution. However when a mixture of the two anions was used, a portion of the homodimers were observed in an asymmetic state of inwardly and outwardly open configurations, underlining the function of a Cl^−^/HCO_3_^−^ exchanger. Similar experiments may allow the study of the outwardly open configuration for SLC26A6 in the future.

## Importance of the elucidation of the SLC26A6 transport mode for understanding the anion transport physiology of the intestine

**FIGURE 4:**
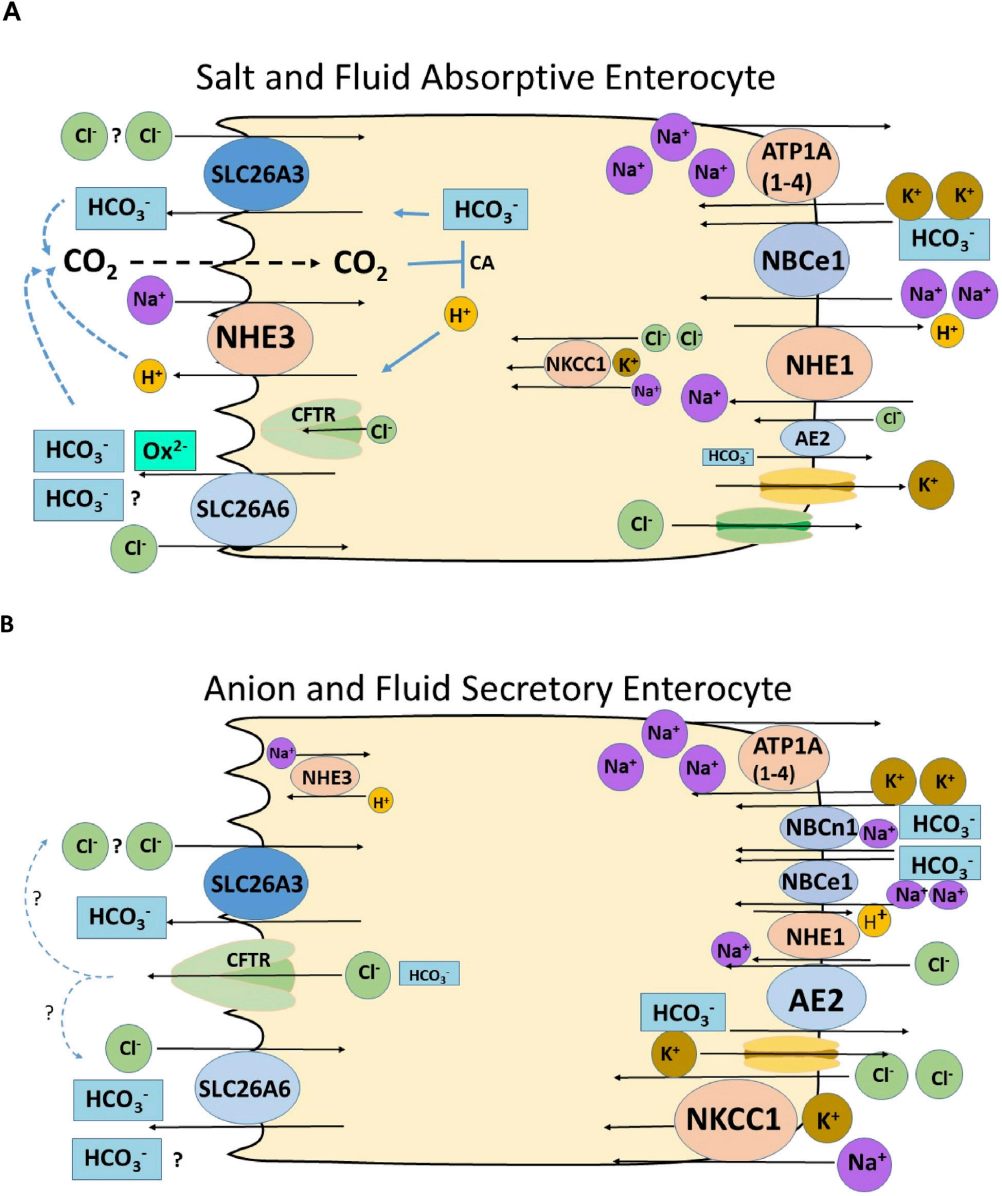
Schematic diagrams of a salt and fluid absorptive and an anion secretory villous enterocyte, depicting the putative role of SLC26A6 in enterocyte anion and fluid absorption and secretion. Most of the immunohistochemical data regarding the location and their trafficking upon stimulation of anion secretion of the transporters are from rodents. **(A)** Villous enterocyte in the salt absorptive mode. The Na^+^/H^+^ exchanger NHE3 and the two Cl^−^/HCO_3_^−^ exchangers SLC26A3 and SLC26A3 are located in the microvillar membrane. NHE3 absorbs Na^+^ in exchange for H^+^, as long as the sodium pump (ATPA (1–4) is active and intracellular Na^+^ concentration is low. The luminal exchange Na^+^/H^+^ exchange process is strongly stimulated by luminal CO_2_, which forms from the exported protons and existing HCO_3_^−^ in the lumen, and results in lowering the pH_i_ of the enterocyte (Xia et al., 2014; Tan et al., 2021). A driving force for the apical Cl^−^/HCO_3_^−^ exchangers may be 1. The increase in subapical HCO_3_^−^, due to the NHE3-driven proton export, 2. A low intracellular Cl^−^ concentration, established by a putative basolateral Cl^−^ export mechanism (KCl cotransporter? Cl^−^ channel?), 3. In the case of a 2HCO_3_^−^/1Cl^−^ exchange mode for SLC26A6 and 2 Cl^−^/1HCO_3_^−^ exchange mode for SLC26A3 (Shcheynikov et al., 2006), the negative membrane potential would favour SLC26A6-mediated 2HCO_3_^−^/1Cl^−^ exchange. Basolateral transporter activity results in an export of Na^+^ and Cl^−^ and an import HCO_3_^−^ via the Na+/H^+^ exchanger NHE1, the Na^+^HCO_3_^−^ cotransporter NBCe1 and possibly also via NBCn1. Water follows the osmotic gradient transcellularly and paracellularly; the latter can be visualized by the widening of the lateral spaces (Larsen et al., 2009). **(B)** Enterocyte in the anion and fluid secretory mode. Although the expression of the anion secretory machinery is expressed much more strongly in the cryptal area, CFTR and the Na^+^K^+^2Cl^−^ cotransporter NKCC1 may also have an overlapping expression with the salt absorptive transporters in some parts of the rodent and human intestine (Jakab et al., 2012b; Tse et al., 2019; Nikolovska et al., 2022; Salari et al., 2023b). While secretory stimuli result in a decrease of microvillar NHE3 abundance, CFTR traffics into the luminal and NKCC1 and NBCe1 into the basolateral membrane (Jakab et al., 2011; Jakab et al. 2012a; Jakab et al. 2012b). AE2 also imports Cl^−^ during anion secretion (Walker et al., 2002). The fast uptake of Cl^−^ from the interstitium prevents a decrease in the intracellular Cl^−^ concentration and cellular shrinkage in the colonic crypt epithelium, abolishing the necessity for luminal Cl^−^ recycling via Slc26a6 and/or Slc26a3 (Bachmann et al., 2003). In the villous epithelium, however, cAMP-induced shrinkage has been reported (Gawenis et al., 2003), and a cell-specific and secretagogue-dependent functional coupling of CFTR and the SLC26 transporters is suggested.

The first investigations on the function of SLC266 were *in vitro* studies in heterologous expression systems and in isolated epithelia. The heterologous expression studies resulted in controversial results, with both electroneutral Cl^−^/HCO_3_^−^ exchange and electrogenic 1Cl^−^/2HCO_3_^−^ exchange described (Ko et al., 2002; Chernova et al., 2005; Alper et al., 2006; Shcheynikov et al., 2006). When the *slc26a6* ^−/−^ mouse became available, the first studies were performed in isolated duodenal and jejunal mucosa in so-called Ussing-chambers, in which the luminal solution is gassed with oxygen and the HCO_3_^−^ output into the lumen is determined by so-called pH-stat microtitration. In this technique, very small additions of acid (in the case of a HCO_3_^−^ secreting epithelium) by a pH-triggered microtitrator keep the luminal pH constant, and the amount of acid moieties added to do so are being recorded (Wang et al., 2005; Tuo et al., 2006). While there was a reduction in basal HCO_3_^−^ output, the short circuit current (Ieq) was not different between *slc26a6* ^−/−^ and wt mucosa. In addition, the HCO_3_^−^ secretory response (ΔJHCO_3_^−^) as well as the ΔIeq to forskolin was not significantly reduced in the absence of Slc26a6. In contrast, in *cftr* ^−/−^ duodenal and jejunal mucosa, both basal HCO_3_^−^ output as well as basal Ieq were significantly reduced, and the ΔJHCO_3_^−^ as well as the ΔIeq response to forskolin were virtually abolished (Seidler et al., 1997). In the case of Slc26a6 operating in a 1Cl^−^ ion uptake against 2 HCO_3_^−^ ions output mode, as postulated, one would expect a difference in the basal Ieq or at least in the forskolin-stimulated ΔIeq between the *slc26a6* ^−/−^ and WT tissue (Figure 4). The results therefore favour a Slc26a6-mediated 1Cl^−^/1HCO_3_^−^ electroneutral exchange. An advantage of the method is that the driving force for HCO_3_^−^ exit across the apical membrane is maximal, because the luminal bath is unbuffered and CO_2_-free, and the rapid gas lift prevents the buildup of unstirred layers. A (hypothetical) caveat is that the absence of CO_2_ in the luminal bath prevents the physiological CO_2_ absorption from the lumen, which membrane-bound and intracellular carbonic anhydrases quickly convert to H^+^ and HCO_3_^−^. The decrease of the subapical pH_i_ due to CO_2_ recycling from the lumen stimulates apical Na^+^/H^+^ exchange, and the increase in HCO_3_^−^ stimulates apical Cl^−^/HCO_3_^−^ exchange (Furukawa et al., 2005).

A method to measure HCO_3_^−^ efflux across the apical membrane without the need to remove the CO_2_/HCO_3_^−^ from the luminal bath was developed by the group of Lane Clarke (Simpson et al., 2005). They mounted murine duodenal mucosa into a perfusion chamber that allowed separate perfusion of the luminal and serosal baths, placed the chamber on the stage of a fluorescence microscope, loaded the villi with the pH-sensitive ratiometric dye BCECF, and measured the change of the pH_i_ upon apical removal and replenishment of Cl^−^ in the luminal bath, while the Cl^−^ concentration in the serosal bath was reduced to prevent the replenishment of Cl^−^ from the basolateral side. Calculation of the actual HCO_3_^−^ flux across the apical membrane requires determination of the intracellular buffer capacity, which is subject to uncertainties in a multicellular tissue layer. Nevertheless, the group used this elegant technique to measure apical Cl^−^/HCO_3_^−^ exchange activity in *slc26a6* ^−/−^ and WT mice (Simpson et al., 2007; Simpson et al., 2010), as well as in *slc26a3* ^−/−^ (Walker et al., 2011) and in *cftr* ^−/−^ mice (Simpson et al., 2005). The data suggest that Slc26a6 imports HCO_3_^−^ during nutrient-induced enterocyte acidification (Simpson et al., 2010), and that membrane depolarisation does not influence Slc26a6-mediated Cl^−^/HCO_3_^−^ exchange rate in the absence of CFTR, supporting the concept of electroneutral transport by Slc26a6 (Walker et al., 2011).

**FIGURE 5:**
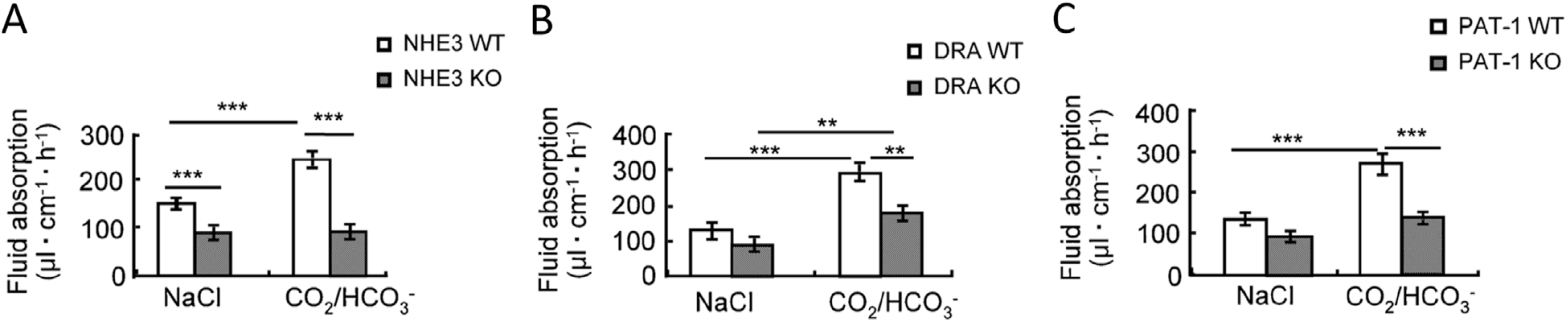
Jejunal fluid absorption rates in **(A)** NHE3-, **(B)** SLC26a3- (DRA) and **(C)** Slc26a6- (PAT-1) deficient mice and their WT littermates. The left bargraphs show the fluid absorption rates in a perfused segment of the upper jejunum in anesthetised WT and gene-deleted mice during perfusion with a prewarmed, unbuffered isoosmolar saline perfusion of pH 7.4. The right bargraphs show the fluid absorption rates after switching the luminal solution to a 95%O_2_/5% CO_2_-gassed, HCO_3_^−^ buffered saline, pH 7.4. CO_2_ enters the enterocyte, lowers the pH_i_, but increases both the intracellular proton as well as the HCO_3_^−^ concentration and stimulates fluid absorption in a NHE3-, DRA- and PAT1-dependent fashion. The subsequent data in the publication show that PAT1 (Slc26a6) presumably imports HCO_3_^−^ in conditions in which enterocyte pH_i_ is acidic (by CO_2_ recycling from the lumen) and the luminal HCO_3_^−^ concentration is high, while SLC26A3 imports Cl^−^, both in the absence and more so in the presence of luminal CO_2_/HCO_3_^−^. See Xia et al. (2014) for more information.

Slc26a6 was clearly involved in jejunal Cl^−^ absorption from the luminal bath in isolated mouse jejunal mucosa, and its deletion also resulted in reduced sodium absorption, suggesting a coupling of the apical NHE3 and SLC26a6 during jejunal salt and water absorption (Seidler et al., 2008). *In vivo* studies supported the role of Slc26a6 in jejunal salt and fluid absorption and, under conditions that acidify the enterocytes, also in HCO_3_^−^ absorption (Singh et al., 2008a; Xia et al., 2014). During single-pass perfusion of corresponding segments of the upper jejunum of anesthetised *slc26a6* ^−/−^ and WT littermates, the fluid absorptive rates were significantly reduced in the slc26a6^−/−^ mice, as was the case in *slc26a3* ^*−/−*^ and in *slc9a3* ^*−/−*^ (*nhe3* ^*−/−*^) mice. However, when unbuffered saline titrated to pH 7.4 prior to entry into the intestinal segment was perfused and the pH of the outflow was measured immediately after exit, the genetic deletion of Slc26a6 resulted in a decrease in jejunal fluid absorption, but not in a decrease in luminal pH, as was the case when Slc26a3 was deleted (Figure 5; Table 1) (Xia et al., 2014). This suggests that, under basal conditions of neutral intracellular and luminal pH, Slc26a6 predominantly exchanges luminal Cl^−^ against another entity than HCO_3_^−^ *in vivo*. This is most likely oxalate, although additional metabolic waste products or metabolic pathway intermediates cannot be excluded (Figure 4 displays hypothetical models of an absorptive and a secretory enterocyte).

**TABLE 1:** Fluid absorption rates and pH-change between inflow and effluent in NHE3-, DRA- and PAT-1-deficient jejunum during single-pass perfusion experiments with unbuffered saline, pH 7.4 While the outflow is significantly more alkaline in *nhe3* ^−/−^ compared to WT jejunum, as expected when an apical proton extrusion mechanism is missing, and is significantly more acidic in *slc26a3* ^−/−^ *(DRA)* compared to WT, as expected when an apical HCO_3_^−^ export mechanism is missing. However, in the *slc26a6* ^−/−^ (*PAT-1*) compared to WT jejunum, the outflow was slightly less acidic. This suggests that Slc26a6 predominantly exchanges extracellular Cl^−^ against an anion other than HCO_3_^−^, most likely oxalate, under the experimental conditions. See Xia et al. (2014) for more information.

		Fluid absorption (μL cm^−1^ h^−1^)	pH change in effluent after perfusion
NHE3	WT	147.7 ± 4.9	−0.29 ± 0.07
	KO	87.3 ± 10.3*	0.27 ± 0.09*
DRA	WT	129.6 ± 10.5	−0.58 ± 0.10
	KO	85.6 ± 5.9*	−1.11 ± 0.07*
PAT-1	WT	145.9 ± 9.0	−0.33 ± 0.05
	KO	107.1 ± 4.9**	−0.14 ± 0.06

*, ** indicates significant difference from respective WT, values.

In contrast, under conditions of a high luminal CO_2_ tension and a high luminal HCO_3_^−^ concentration, for example, after the alkaline pancreatic juice enters the acidic lumen of the duodenum after a meal, Slc26a6 may even import HCO_3_^−^ into the enterocyte, although we do not know which intracellular anion is exchanged, possibly Cl^−^ in addition to Ox^2−^, depending on the concentration gradients for the exchangeable anions across the apical membrane (Xia et al., 2014). Indeed, electrogenic oxalate secretion is favoured by the high affinity of oxalate to Slc26a6 (Jiang et al., 2002) and the negative membrane potential of the enterocytes. These *in vivo* data in the slc26a6-knockout mouse confirmed earlier *in vitro* data showing that Slc26a6 may function as an oxalate secretory mechanism across the small intestinal mucosa (Knauf et al., 2011). Indeed, *slc26a6* ^−/−^ mice on an oxalate-rich diet develop oxalate kidney stones (Jiang et al., 2006).

In contrast, deletion of Slc26a3, another Cl^−^/HCO_3_^−^ exchanger expressed in the brush border membrane of enterocytes, which has a similar expression level and villous-predominant distribution as Slc26a6 in the upper small intestine (Liu et al., 2015; Seidler and Nikolovska, 2019), resulted in an approximately equal reduction of jejunal fluid absorption *in vivo* than the deletion of Slc26a6, but also resulted in a strong acidification of the effluent of the perfused jejunal segment (Table 1; Xia et al., 2014). Alkalinisation following luminal acid challenge in the duodenum *in vivo* was also predominantly dependent on SLC26a3 and CFTR expression, but not on Slc26a6 expression (Singh et al., 2008b; Singh et al., 2013). This suggests that in the mouse *in vivo*, Slc26a3 is the major transport mechanism for luminal alkalinisation in both the small and large intestine in the basal state and after a luminal acid load, but that both Slc26a3 and Slc26a6 are involved in small intestinal fluid absorption. The predominant transport mode for Slc26a3 in the mouse small intestine *in vivo* appears to be electroneutral Cl^−^/HCO_3_^−^ exchange and that of Slc26a6 to be electrogenic Cl^−^/Ox^2−^ exchange, but the latter may be complemented by electroneutral Slc26a6-dependent Cl^−^/HCO_3_^−^ exchange depending on the acid/base status of the lumen the blood and the epithelium (Figure 4). Human SLC26A3 also acts as a major alkalinization mechanism in the human ileum and colon (Holmberg et al., 1975). The role for SLC26A6 is the human gut is not well known, but may at least in part also be anion/oxalate exchange, based on the recent findings about a human SLC26A6 transport defect (Figure 4) (Corniere et al., 2022). Why this is so is presently not clear, given the fact that current data favour an electroneutral Cl^−^/HCO_3_^−^ exchange for both human and murine Slc26a6 and Slc26a3 orthologs, an affinity for oxalate for both transporters, and a similar localisation for Slc26a6 and Slc26a3 in the small intestine.

Taken together, a role for SLC26A6 in small intestinal Cl^−^ absorption, and, under situations of high intracellular HCO_3_^−^ concentration also HCO_3_^−^, has been established *in vitro* and *in vivo*, and the counterions may be oxalate as well as HCO_3_^−^. The role of Slc26a6 for secretagogue-stimulated mall intestinal HCO_3_^−^ and fluid secretion is less well established. CFTR-mediated Cl^−^ secretion into the lumen of the intestinal crypts and by the high CFTR expressor cells in the rat and human jejunal villi leads to a rapid increase in the uptake of Cl^−^ via the basolateral uptake mechanisms NKCC1 and AE2. This process is enhanced by trafficking of CFTR and NKCC1 into the apical and basolateral membrane, respectively (Jakab et al., 2011; Jakab et al., 2012a; Jakab et al., 2012b). Basolateral Cl^−^ uptake by AE2 will decrease the intracellular HCO_3_^−^ concentration and favour Cl^−^ secretion over HCO_3_^−^ secretion via CFTR. A CFTR-dependent Cl^−^ recycling via apical Cl^−^/HCO_3_^−^ exchangers Slc26a6 and Slc26a3 may occur to a minor degree in the mouse small intestinal villous area and colonic cryptal mouth area, but the majority of stimulation-associated HCO_3_^−^ secretion, which is always much lower than the stimulation-associated Cl^−^ secretion, is probably due to the bicarbonate permeability of the CFTR channel (Figure 4). Slc26a6 has been reported to traffick into the rat proximal colonic brush border membrane (BBM) upon cAMP-dependent stimulation by lubiprostone, while Slc26a3 was internalized (Jakab et al., 2012b). Clearly, uncertainties related to antibody specificity, potential species differences, segmental differences that have not been unraveled completely, and as yet incomplete differentiation of intestinal organoids *in vitro* hamper the applicability of the schematic cartoons constructed for Figure 4, which summarize functional, molecular biological and immunohistochemical data from many different reports. More structure-function studies and the new organoid technology may pave the way for rapid progress in understanding the remaining questions related to the role and the molecular mechanisms of SLC26A6 in intestinal acid-base transport.

## Controversies related to the role of Slc26a6 in pancreatic HCO_3_^−^ secretion

In the first description of SLC26A6 cloning and antibody generation, an immunohistochemical image of human pancreatic ducts was shown, with apical staining with an anti-SLC26A6 antibody (Lohi et al., 2000). mRNA expression for SLC26A6 was also found high in human pancreatic tissue. Subsequently, *in vitro* studies were performed in isolated pancreatic ducts from *slc26a6* ^−/−^ and WT mice (Wang et al., 2006; Ishiguro et al., 2007; Song et al., 2009) and guinea pigs (Stewart et al., 2009; Stewart et al., 2011). A sophisticated combination of electrophysiological and fluorometric techniques during ion substitution and inhibitor applications was used to study the electrogenicity of Cl^−^/HCO_3_^−^ exchange across the luminal membrane of mouse interlobular ducts during cAMP stimulation (to open CFTR channels), and the results were interpreted as a demonstration of a 2 HCO_3_^−^/1 Cl^−^ coupling of Slc26a6 and, in its absence, the appearance of a 1 HCO_3_^−^/2Cl^−^exchanger in the luminal membrane, probably Slc26a3 (Song et al., 2012). Although the results were carefully contained, I see alternative explanations for the data, based on scientific advances made in the last decade. Firstly, the pancreatic ductal epithelium, particularly of humans and guinea pigs, expresses high levels of CFTR in the luminal membrane, but low if any levels of the Na^+^K^+^2Cl^−^ cotransporter NKCC1 in the basolateral membrane, in contrast to the situation in small intestinal and colonic crypts, which strongly express both CFTR and NKCC1 (Jakab et al., 2011; Yamaguchi et al., 2017; Salari et al., 2023a). Secondly, while intestinal crypts and, to a lesser extent, villous cells also express basolateral AE2, which can serve as an alternative Cl^−^ uptake mechanism in exchange for intracellular HCO_3_^−^, the rat pancreatic duct cells express basolateral Na^+^HCO_3_^−^ cotransporters and Na^+^/H^+^ exchangers, but do not express AE2 (Roussa et al., 2001). The situation in the human and guinea pig pancreatic ducts, which secrete an isotonic Na^+^(K^+^)-HCO_3_^−^ solution, is completely different from that in the intestine, because the basolateral membrane lacks the Cl^−^ absorptive transporters NKCC1 and AE2, and the strongly expressed basolateral NBCe1 favours the uptake of HCO_3_^−^, but not of Cl^−^ (Ishiguro et al., 2012). Low intracellular Cl^−^ stimulates the WNK pathway in pancreatic duct cells, resulting in an increase in the HCO_3_^−^ conductance of the CFTR channel (Park et al., 2010). Recent computational modelling of the composition of ion transporters in the guinea pig (and presumably human) pancreatic ducts suggested that in the proximal small ducts, Slc26a6 exchanges luminal Cl^−^ against intracellular HCO_3_^−^ with either 1:1 or 1:2 coupling, but that the rising intraductal HCO_3_^−^ and decreasing Cl^−^ concentrations rapidly inhibit this exchange, and HCO_3_^−^ is secreted via the CFTR channel. In order to achieve an isomolar cation-HCO_3_^−^ concentration in the distal pancreatic duct, low activity of basolateral Cl^−^ uptake pathways is a key requirement. This is in line with current evidence (Yamaguchi et al., 2017). A recent publication investigated the contribution of Slc26a6 to stimulated pancreatic juice secretion by the *ex vivo* mouse pancreas and found that the genetic deletion of Slc26a6 reduced the *ex vivo* fluid secretory rate by approximately 35% (Munemasa et al., 2019). The contribution to pancreatic fluid secretion was HCO_3_^−^-dependent. The authors also concluded that another major HCO_3_^−^-independent pathway is the primary driver of the fluid secretion process in the mouse pancreas. Interestingly, they localised Slc26a6 to the apical membrane of the pancreatic acini (Munemasa et al., 2019). This study also suggests that the other Slc26 members that mediate Cl^−^/HCO_3_^−^ exchange and have been found expressed in pancreatic ducts (Andharia et al., 2018), are likely to be of minor importance for pancreatic ductal secretion in the mouse.

## Search for disease-causing variants in the human SLC26A6 gene

In contrast to mutations in SLC26A2, SLC26A3, and SLC26A4, that have been cloned during the search for the molecular cause of hereditable diseases in humans, variants in *SLC26A6*, although sought for in databases with genetic information on patient groups, have not been associated with human diseases until recently. An obvious search focus was on patient cohorts with pancreatic disease. However, no associations were found between the incidence of chronic pancreatitis and potentially disease causing variants in SLC26A6 (Balazs et al., 2015). At the time, this was surprising because Slc26a6 has been described as essential for CFTR-dependent pancreatic HCO_3_^−^ secretion (Wang et al., 2006) and because the association of CFTR variants, even clinically mild ones, or those which only affected the CFTR-dependent HCO_3_^−^ transport, with chronic idiopathic pancreatitis have been found in several cohorts (Bernardino et al., 2003; Lee et al., 2003; Weiss et al., 2005; LaRusch et al., 2014; Berke et al., 2022). One explanation may be that the rodent pancreas is not an ideal model for the assessment of human pancreatic fluid dynamics, as the rodent pancreas is capable of sustaining relatively high fluid secretory rates in the absence of HCO_3_^−^ (Munemasa et al., 2019). In addition, the rodent pancreas does not develop severe pancreatic disease in the absence of CFTR expression, possibly due to the presence of alternative Cl^−^ channels (Clarke et al., 1994). In contrast, human pancreatic fluid secretion, like that of the guinea pig, is strongly dependent on HCO_3_^−^ availability and the ability of the CFTR channel to mediate HCO_3_^−^ secretion.

## First description of a genetic defect in SLC26a6 in a patient with enteric hyperoxaluria and kidney stones

**FIGURE 6:**
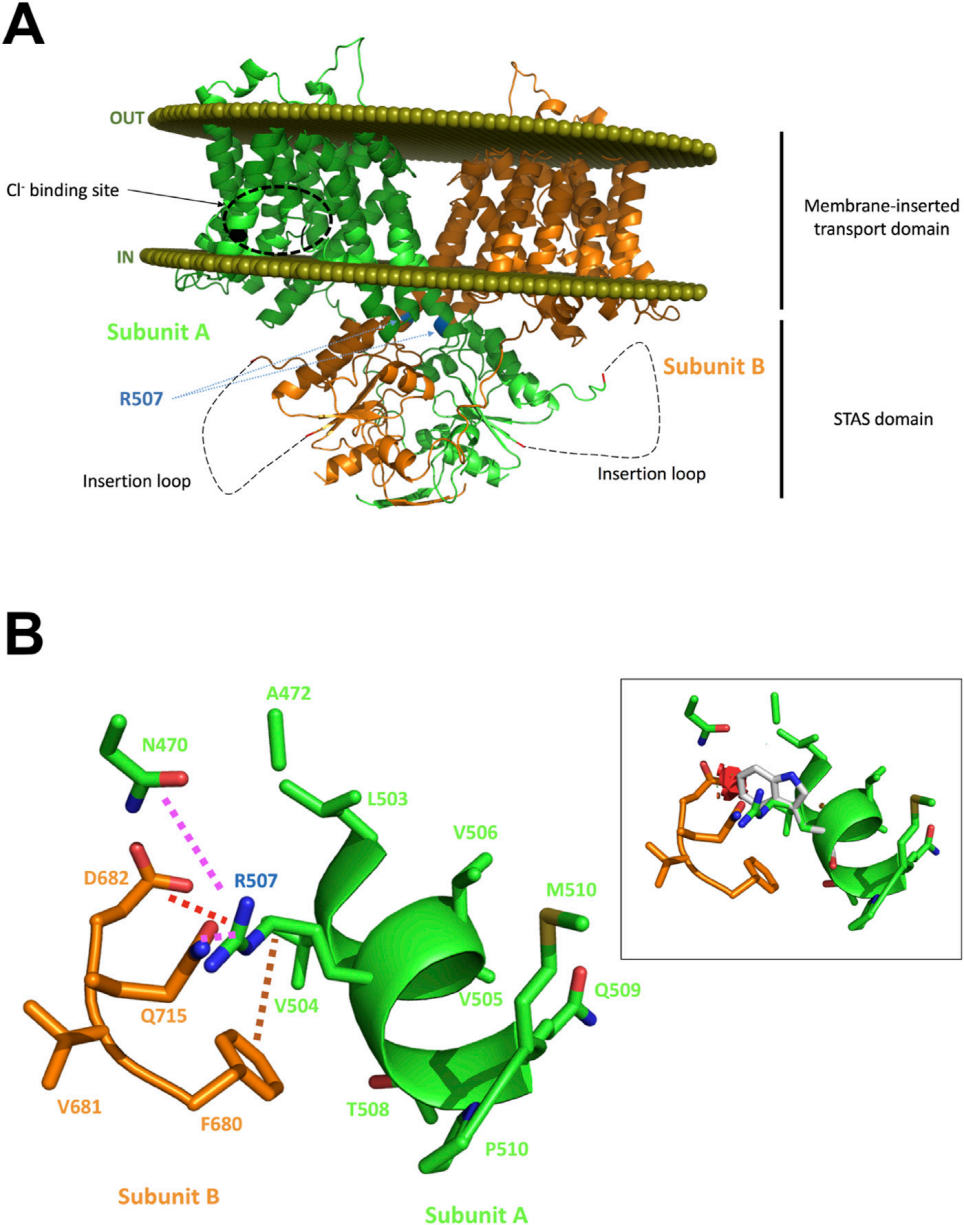
In silico analysis of the putative effects of the R507W substitution on the SLC26A6 protein structure. Panel **(A)**: Structural model of the human SLC26A6 protein. The two subunits are presented with one subunit colored in green and the other in orange (cartoon representation). The olive spheres represent the boundary of the lipid bilayer as computed with the PPM server. A zone predicted to interact with the Cl^−^ ion as proposed in the mouse Slc26a9 experimental structure is highlighted in subunit A for orientation. R507 residues of both subunits are flagged; this residue is located on the last helix of the transmembrane domain. The insertion loop that could not be built is shown as a dashed line. The mutation is expected to destabilize the protein and to impede optimal insertion into the lipid bilayer. Panel **(B)**: Analysis of the region surrounding R507. R507 makes several hydrogen bonds with it surrounding (magenta dashed lines), form a salt bridge with D682 (red dashed line) and has hydrophobic/aromatic contacts (brown dashed line) with, for example, F680. Panel **(B)**, inset: Attempts to replace R507 on the computer screen by a tryptophan generate multiple steric clashes, illustrated here by several red disks. Alternative orientations of the W side chain would also create even more severe clashes, and certainly impede appropriate interaction with the other subunit, damage the oligomeric structure and the helical structure and could indirectly affect proper orientation/association with the membrane. See Corniere et al. (2022) for more information.

A second focus for exploring the relevance of SLC26A6 mutations has been on kidney stone cohorts, or cohorts with a high risk of developing nephrolithiasis, because of the importance of Slc26a6 for intestinal secretion of oxalate in mice and the high affinity of oxalate for the human SLC26A6 protein. However, the first studies did not show an association between SLC26A6 variants and hyperoxaluria/hyperoxalemia or the risk of nephrolithiasis in these cohorts (Monico et al., 2008; Corbetta et al., 2009; Scherer et al., 2023). Recently however, a young female patient with very severe recurrent oxalate nephrolithiasis was identified in the Department of Metabolic and Renal Diseases of the University hospital in Amiens, France (Corniere et al., 2022). Biochemical analysis of the stone revealed it to be calcium oxalate. Urine analysis showed hyperoxaluria and mild hypocitraturia, and dietary history showed very low calcium intake but no excessive intake of oxalate-rich food. Whole exon sequencing identified a rare heterozygous missense mutation (c.1519C > T/p.R507W) in the SLC26A6 gene, but no mutations in other risk genes for oxalate nephrolithiasis. The father carried the same mutation and showed hyperoxaluria but not hypocitraturia and a very high daily fluid intake which may have prevented the formation of oxalate kidney stones. WT and the mutant SLC26A6 was expressed in OKP (opossum kidney) cells and the effect of the p.R507W mutation on the expression and function of human SLC26A6 was investigated. The mutant was less stable in the membrane and showed a reduced transport rate for Cl^−^/Ox^2−^ exchange compared to WT Slc26A6. Interestingly, cotransfection studies showed strong dominant-negative effects of the mutant on the wild-type protein. This may explain why a heterozygous mutation results in a severe phenotype. In silico analysis of the mutant, based on the then published SLC26A9 structure (that of SLC26A6 not having been published yet), suggested that the mutation was situated in the last helix of the transmembrane domain close to the cytoplasmic STAS domain and may interfere with the optimal insertion of the SLC26A6 protein into the plasma membrane (Figure 6). This is the first strong evidence that mutations in the *SLC26A6* gene that result in a functionally defective SLC26A6-mediated oxalate transport are strong risk factors for the development of oxalate nephropathy. Additional dietary factors were required for the stone phenotype, one of these factors being the low Ca^2+^ diet of this patient, since the change to a Ca^2+^-rich diet and sufficient fluid intake prevented further stone formation. The same is true for slc26a6^−/−^ mice, which develop nephrolithiasis during high oxalate uptake, but not with a low oxalate diet (Seidler and Nikolovska, 2019).

Other groups have also found polymorphisms in the *SLC26A6* gene in kidney stone cohorts, but the functional significance of the individual polymorphisms for oxalate transport has not been elucidated in the same detail as in the study discussed above, and therefore the functional involvement of these variants in the development of the nephrolithiasis of these patients not certain (Monico et al., 2008; Corbetta et al., 2009; Lu et al., 2016). An interesting study from Israel also reported two polymorphisms in the STAS domain of SLC26A6 in two heterozygous carriers. These patients both had low urinary citrate concentrations (also a major risk factor for the development of Ca^2+^oxalate kidney stones), but only one patient developed Ca^2+^ oxalate stone. SLC26A6 mutants corresponding to the observed variants of the two patients were constructed by site-directed mutagenesis, expressed and functionally characterised in HEK293 cells. The one homolog mutation of the patient that had developed Ca^2+^ oxalate stones abolished the expression and function of SLC26A6 and impaired the regulation of SLC13-mediated citrate transport by SLC26A6. The SLC26A6 variant of the second patient showed reduced SLC26A6 protein expression and membrane trafficking, retained full transport activity and impaired the regulation of the citrate transporter (Shimshilashvili et al., 2020). The data follow on earlier work in heterologous expression systems and in mice that described a regulatory effect of SLC26A6 on the sodium-dicarboxylate cotransporter NaDC-1, which primarily mediates the co-transport of Na+ and tricarboxylic acid cycle intermediates, such as citrate and succinate (Ohana et al., 2013; Khamaysi et al., 2019). Taken together, the results from mutation screening of patient cohorts demonstrate the variable impact that SLC26A6 mutations may have on enteric and/or renal transport physiology. Future investigations of the anion transport defect (if any) associated with additional SLC26A6 mutations, by similar site directed mutagenesis and structure function studies as performed by Corniere et al., and Shimshilashvili et al., may broaden the spectrum of SLC6A6-associated human diseases.

## Pharmacological agents to influence SLC26A6 transport rate

One major experimental problem in unraveling the contributions of different anion transporters to a given physiological event was the lack of specific inhibitors. Both the chloride channels blockers, such as NPPB, and the anion transport inhibitors, such as the stilbene derivatives, were fairly nonspecific, inhibiting some, but not all, members of the SLC4 and SLC26 gene family, as well as some Cl^−^ channels, but also less closely related biological structures such as the mitochondrial permeability transition pore (Boron, 2001; Sanders et al., 2012; Liu et al., 2016). Accordingly, nonwanted and nonspecific effects are problematic for the interpretation of the experimental data. Therefore, elucidating the regulation of SLC26a6 in the epithelia that endogenously express the transporter has been challenging, given the fact that epithelia may express different SLC26 isoforms in the same cell and even the same membrane. The few things that we know about the physiologic significance of endogenous Slc26a6 have been generated in *slc26a6* ^−/−^ mice (described and cited in the previous paragraphs), or in genetically manipulated cell lines with endogenous SLC26A6 expression (Freel et al., 2009; Anbazhagan et al., 2019; Arvans et al., 2023). In contrast, immunohistochemical data are often from rat intestine, because nonspecific effects are less problematic with polyclonal antibodies generated in rabbits, and monoclonal antibodies generated in mouse. In addition, the *slc26a6* ^−/−^ mouse may have adapted to the loss of this transporter by upregulation of other anion transport pathways, thus obscuring its biological significance.

**FIGURE 7:**
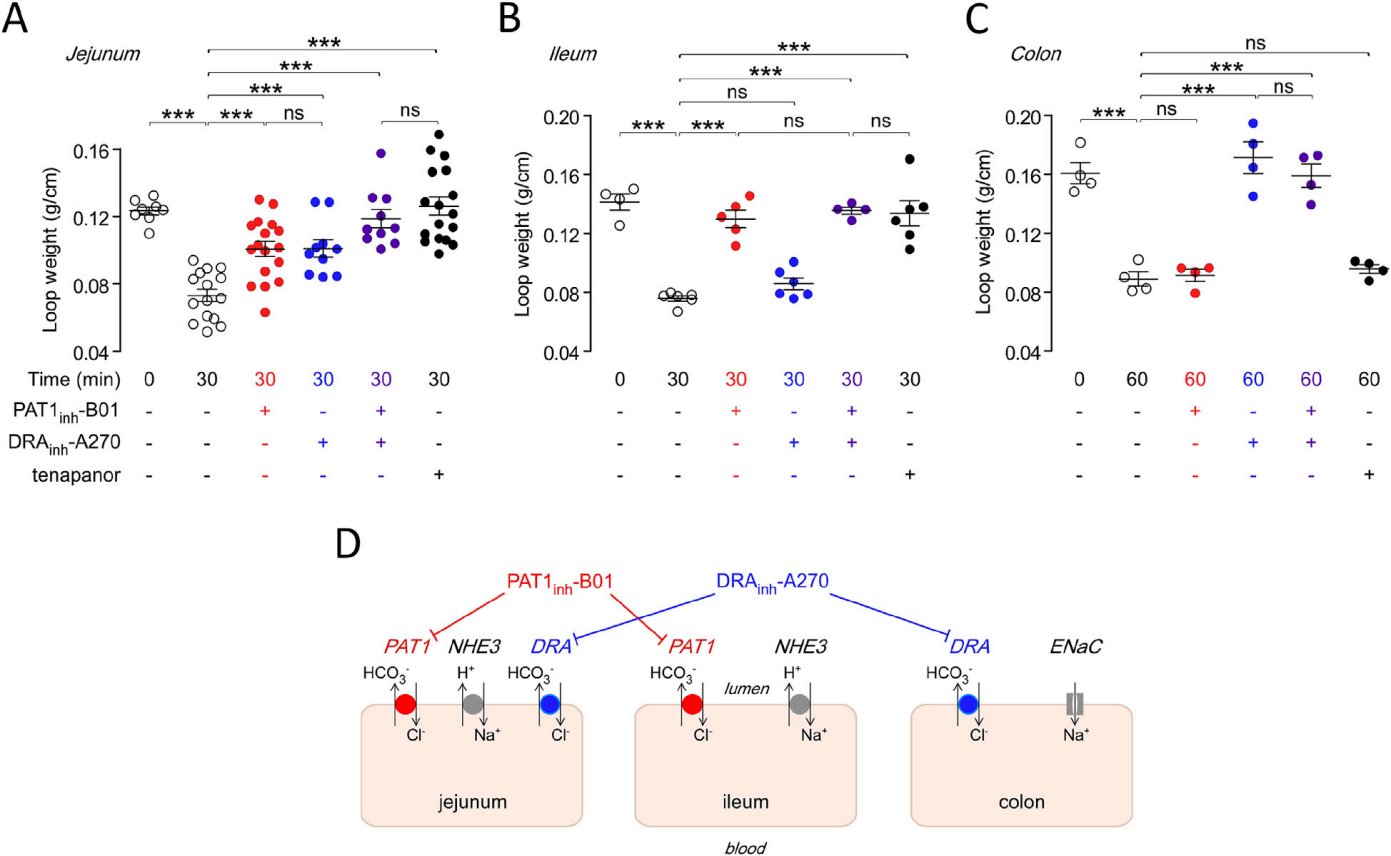
Region specific effects of the inhibitors for Slc26a6 (PAT1), Slc26a3 (DRA) and Slc9a3 (NHE3) in the mouse intestine. Upper panel shows the effect of luminal PAT1inh-B01 (30 μM), DRAinh-A270 (10 μM), and tenapanor (10 μM), individually and together, on loop weight-to-length ratio at 30 min in mouse **(A)** midjejunal closed loops, **(B)** ileal closed loops, and **(C)** distal colonic closed loops. **(D)** Cartoon schematically displays the differential effect of the inhibitors for PAT1, DRA and NHE3 in the mouse intestine. See Cil et al. (2021) for further details.

Recently, specific inhibitors have been generated for several Cl^−^ channels, as well as for members of the SLC26 family, including SLC26A6. In 2021, the group of Alan Verkman first described the identification of a highly selective SLC26A6 inhibitor by high throughput screening, using as a readout the inhibition of Cl^−^ efflux, in exchange for iodide, from anion-transporter-transfected FRT cells by fluorometric assessment of YFP quenching (Cil et al., 2021). A concentration that fully inhibited SLC26a6-mediated Cl^−^ efflux did not affect Cl^−^ efflux from FRT cells transfected with SLC26A3, A4, A9, and TMEM16a, and also did not affect the CFTR-mediated Isc-response to forskolin in a bronchial cell line. The group also tested the compound, named PAT1_inh_-B01, as well as the SLC26A3 (DRA)–inhibitor DRA_inh_-A270, in the murine intestine using an *in vivo* closed loop model, in which different segments of the intestine are tied off, filled with drug or vehicle-containing NaCl solution, excised and weighted after a certain time period. These results suggested that Slc26a6 was invovled to a similar degree in salt and water absorption as Slc26a3 in salt and water absorption as Slc26a3 in the murine jejunum, but was the major Cl^−^ absorption pathway in the ileum, while Slc26a3 was the major absorption pathway in the distal colon (Figure 7). The group also tested the efficacy of PAT1_inh_-B01 in the ileum of mice with a F508del mutation in the CFTR gene, and found that the inhibitor completely blocked NaCl- and fluid absorption in this segment of the *F508del* mutant mouse, as did the NHE3 inhibitor tenapanor, whereas the Slc26a3-inhibitor DRAinh-A270 was without effect. The authors concluded that Slc26a6 and Slc26a3 mediate Cl^−^ and water absorption in the murine intestine in a segment-specific manner. Based on the selective role of SLC26a6 in ileal fluid absorption in both WT and F508del mice, the authors speculate that the drug may be beneficial for the small intestinal obstructive problems in patients with cystic fibrosis. A more recent publication presented data on a more potent, also selective Slc26a6 inhibitor name PAT1inh-A0030 (Chu et al., 2023). The availability of these inhibitors may greatly facilitate the elucidation of the physiological role of Slc26a6 in cellular and organ function. However, a caveat against its long-term use, in particular in the kidney stone prone CF patient population, may come from a study by Xia et al. (2014), who studied the fluid absorptive and HCO_3_^−^ secretion rates in anesthetised Slc26a6-, Slc26a3, NHE3-and NHE2-deleted mice by single-pass perfusion. They also found similar reductions in jejunal fluid absorption in *slc26a6* ^−/−^ and *slc26a3* ^−/−^ mice, as did Cil et al. (2021). However, a reduction in luminal HCO_3_^−^ output, as expected for the deletion of an apical Cl^−^/HCO_3_^−^ exchanger, was only observed in *slc26a3* ^−/−^ mice. This suggests that murine jejunal Slc26a6 exchanges luminal Cl^−^ (because that was the only anion present in the luminal perfusate) for an anion other than HCO_3_^−^. The most likely candidate is oxalate (Jiang et al., 2002). The high oxalate but low Cl^−^ affinity of the human compared to the mouse SLC26A6 paralogue suggests that the inhibition of intestinal SLC26A6 in human may increase the risk of hyperoxalemia in individuals at risk (Clark et al., 2008). On the other hand, if SLC26A6 does indeed predominantly exchanges luminal Cl^−^ for HCO_3_^−^ in the human ileum, its inhibition may be equally or more beneficial for prevention of distal ileal obstruction (DIOS) than the inhibition of NHE3-mediated luminal Na^+^/H^+^ exchange, which has been shown to both inhibit fluid absorption and increase HCO_3_^−^ output in the small and large intestine in CFTR-deficient mice (Tan et al., 2021), as well as reduce the risk of intestinal obstructions in these mice when given orally for 3 weeks (Tan et al., 2022), and for which the intestine-specific, FDA-approved inhibitor tenapanor is available (Dominguez Rieg and Rieg, 2024). However, it has also been demonstrated in murine intestine and in cell culture, that NHE3 is involved in exporting protons that are absorbed by the proton-coupled peptide (PEPT-1), amino acid transporter SLC36A1, and short chain fatty acid absorbing monodecarboxylate transporter MCT-1, which all acidify the enterocyte during the absorption of the respective nutrient that they transport, raising concerns about negative effects of NHE3-inhibition on nutrient absorption (Tamai et al., 1995; Thwaites et al., 2002; Inigo et al., 2006; Chen et al., 2010). On the other hand, CF patients whose functional defect in the CFTR protein is amenable to CFTR-targeted therapy have shown positive effects on weight development by CFTR-corrector/potentiator therapy, suggesting that multiple options may soon be available to treat the defects in intestinal function in CF patients (Guimbellot et al., 2019; Regard et al., 2023). Careful observation on nutrient balance will be necessary when treating CF patients with inhibitors for acid/base and electrolyte transport in the intestine.

## Conclusions and future areas for research

The multifunctional anion exchanger SLC26A6 was cloned 25 years ago, but its exact localisation, transport regulation and physiological significance at the organ, cellular and subcellular level are still being elucidated, and controversial data exist in the literature. In recent years, there have been major advances in the detailed understanding of the physiological functions of SLC26A6: Firstly, the elucidation of its structure at high resolution and the subsequent structure-function studies help to resolve the controversies regarding the transport mode of the different anions and allow a better interpretation of the published physiological experiments. Secondly, the first detailed functional analysis of a disease-causing SLC26A6 mutation detected in the genome of a patient with severe hyperoxaluria and nephrolithiasis has recently been described. Finally, selective inhibitors of SLC26A6 transport function have been identified. The new knowledge, combined with new tools, will pave the way for more detailed structure-function analyses andn more insight into the physiological role and pathological consequences of genetic defects in SLC26A6. Specific anti-SLC26A6 antibodies or/and an epitope-tagged SLC26A6 knock in mouse are urgently needed to gain insight into the cellular localisation of SLC26A6 throughout the body, to analyse its transport regulation and to find ways to target SLC26A6 for therapy.
